# Regulator of calcineurin 1 differentially regulates TLR-dependent MyD88 and TRIF signaling pathways

**DOI:** 10.1371/journal.pone.0197491

**Published:** 2018-05-25

**Authors:** Zheng Pang, Robert D. Junkins, Renee Raudonis, Adam J. MacNeil, Craig McCormick, Zhenyu Cheng, Tong-Jun Lin

**Affiliations:** 1 Department of Pathology, Dalhousie University, Halifax, Nova Scotia, Canada; 2 Department of Microbiology and Immunology, Dalhousie University, Halifax, Nova Scotia, Canada; 3 Beatrice Hunter Cancer Research Institute, Halifax, Nova Scotia, Canada; 4 Department of Pediatrics, IWK Health Centre, Halifax, Nova Scotia, Canada; University of Tennessee Health Science Center, UNITED STATES

## Abstract

Toll-like receptors (TLRs) recognize the conserved molecular patterns in microorganisms and trigger myeloid differentiation primary response 88 (MyD88) and/or TIR-domain-containing adapter-inducing interferon-β (TRIF) pathways that are critical for host defense against microbial infection. However, the molecular mechanisms that govern TLR signaling remain incompletely understood. Regulator of calcineurin-1 (RCAN1), a small evolutionarily conserved protein that inhibits calcineurin phosphatase activity, suppresses inflammation during *Pseudomonas aeruginosa* infection. Here, we define the roles for RCAN1 in *P*. *aeruginosa* lipopolysaccharide (LPS)-activated TLR4 signaling. We compared the effects of *P*. *aeruginosa* LPS challenge on bone marrow-derived macrophages from both wild-type and RCAN1-deficient mice and found that RCAN1 deficiency increased the MyD88-NF-κB-mediated cytokine production (IL-6, TNF and MIP-2), whereas TRIF-interferon-stimulated response elements (ISRE)-mediated cytokine production (IFNβ, RANTES and IP-10) was suppressed. RCAN1 deficiency caused increased IκBα phosphorylation and NF-κB activity in the MyD88-dependent pathway, but impaired ISRE activation and reduced IRF7 expression in the TRIF-dependent pathway. Complementary studies of a mouse model of *P*. *aeruginosa* LPS-induced acute pneumonia confirmed that RCAN1-deficient mice displayed greatly enhanced NF-κB activity and MyD88-NF-κB-mediated cytokine production, which correlated with enhanced pulmonary infiltration of neutrophils. By contrast, RCAN1 deficiency had little effect on the TRIF pathway *in vivo*. These findings demonstrate a novel regulatory role of RCAN1 in TLR signaling, which differentially regulates MyD88 and TRIF pathways.

## Introduction

Toll like receptors (TLRs) are a family of transmembrane receptors that recognize diverse molecular patterns derived from microbes [1]. Upon binding of ligands, TLRs dimerize and recruit adapter proteins to their cytoplasmic Toll/IL-1 receptor domain to initiate downstream signaling [2]. TLR4 binding to lipopolysaccharide (LPS) activates two distinct signalling pathways; the myeloid differentiation primary response 88 (MyD88) pathway, and the Toll/IL-1R domain—containing adapter inducing IFN-β (TRIF) pathway [3]. MyD88-dependent signal transduction activates NF-κB via phosphorylation and ubiquitin-mediated degradation of its inhibitory protein IκBα, which allows NF-κB nuclear translocation and transactivation of a multitude of proinflammatory cytokines and chemokines, including IL-6, TNF and macrophage inflammatory protein (MIP)-2 [2, 4]. In the parallel TRIF-dependent pathway, LPS activates tank-binding kinase-1 (TBK1) and I-kappa-B kinase epsilon (IKKε), leading to phosphorylation and activation of interferon regulatory transcription factor 3 (IRF3) and IRF7 [5, 6]. Activated IRF3 and IRF7 drive transcription of interferon-α (IFNα) and interferon-β (IFNβ), and the chemokines RANTES (Regulated on Activation, Normal T cell Expressed and Secreted) and IP-10 (Interferon γ-inducible Protein 10) [7, 8]. Both MyD88- and TRIF-dependent pathways have been found to contribute to host defense against the microbial infection [9–12].

TLR signaling is tightly regulated. Unrestrained production of proinflammatory mediators through TLR signaling can disrupt the balance between pro- and anti-inflammatory responses and cause severe inflammatory and autoimmune diseases [13, 14]. Many negative regulators of TLR signaling have been identified in the past decade [15]. We previously identified regulator of calcineurin 1 (RCAN1) as a central negative regulator of inflammation during *P*. *aeruginosa* infection *in vivo*; RCAN1-deficient mice displayed aberrant NF-κB activation and increased levels of inflammatory cytokines, which correlated with increased mortality [16]. RCAN1 has not yet been linked to regulation of TLR signaling.

The *RCAN1* gene is located on chromosome 21 in the Down syndrome critical region, and is highly expressed in various tissues including brain, heart, muscle, liver, kidney, lung and testis [17–19]. It has seven exons which can be alternatively spliced to render 4 different transcript isoforms (*RCAN1-1*, *RCAN1-2*, *RCAN1-3* and *RCAN1-4*) [18]. RCAN1 was previously shown to inhibit calcineurin phosphatase activity by direct interaction with the catalytic subunit of calcineurin, leading to suppression of nuclear factor of activated T cells (NFAT) activation and signaling axis [20].

In this study, we used LPS from *P*. *aeruginosa*, a potent activator of TLR4, to examine the role of RCAN1 in both MyD88- and TRIF-dependent signaling *in vivo* and *in vitro*. We found that RCAN1 deficiency significantly enhances MyD88-NF-κB-mediated cytokine production (IL-6, TNF and MIP-2) and NF-κB activity both *in vivo* and *in vitro*. Moreover, we found that RCAN1-deficient macrophages display increased IκBα phosphorylation and no significant change on IκB kinase (IKK) α/β phosphorylation compared to wild-type macrophages. By contrast, RCAN1 deficiency downregulates the interferon-stimulated response elements (ISRE)-mediated cytokine production (IFNβ, RANTES, IP-10) and TRIF-IRF7-ISRE pathway activation *in vitro*. Interestingly, RCAN1 deficiency has limited effects on the TRIF-IRF-ISRE pathway *in vivo*. These findings suggest that RCAN1 is a negative regulator of the TLR-MyD88-NF-κB signaling pathway through targeting IκBɑ, and to our knowledge, provide the first line of evidence that RCAN1 plays a role in mediating TLR-TRIF-IRF7-ISRE signaling pathway activation.

## Materials and methods

### Animals

RCAN1-deficient mice were generated as described previously with a deletion of exons 5 and 6 leading to deficiency of *Rcan1* products (Rcan1-1 and Rcan1-4) [21], and were provided by J. Molkentin (Cincinnati Children’s Hospital Medical Center, University of Cincinnati, Cincinnati, OH). C57BL/6 mice were purchased from Charles River Laboratories and were used as wild-type controls. All animal protocols were approved by the University Committee on Laboratory Animals, Dalhousie University, in accordance with guidelines of the Canadian Council on Animal Care.

### Antibodies

Antibodies for phospho-IκBα (2859), total IκBα (9242), phospho-IKKα/β (2697), total IKKα (2682), total IKKβ (2684), phospho-ERK (9101), total ERK (9102) and phospho-p38 (9211) were purchased from Cell Signaling. Antibody for IRF7 (ab109255) was purchased from Abcam. Antibodies for total p38 (sc-535), IRF3 (sc-9082), IRF7 (sc-9083), actin (sc-1616) and all secondary antibodies were purchased from Santa Cruz Biotechnology.

### Lung stimulation with *P*. *aeruginosa* LPS and collection of lung and bronchoalveolar lavage fluid (BALF)

*P*. *aeruginosa* LPS (L8643) was purchased from Sigma-Aldrich. Mice were intranasally administered with 1 μg *P*. *aeruginosa* LPS per gram of body weight for 4 h or 24 h. After stimulation, BALF was obtained by lavage of lungs with 1 ml phosphate-buffered saline (PBS) containing soybean trypsin inhibitor (100 μg/ml). Lung tissues were obtained for histology study, detection of cytokines and myeloperoxidase (MPO) assay. Briefly, lung tissues were homogenized in 50 mM HEPES buffer (4 μl/mg lung) containing soybean trypsin inhibitor (100 μg/ml). Lung homogenates were centrifuged at 4°C for 20 min at 18,000 x *g*. The supernatants were stored at −80°C for later cytokine analysis. The pellets were resuspended and homogenized in 0.5% cetyltrimethylammonium chloride (4 μl/mg lung) and centrifuged as described above. The cleared extracts were used for MPO assay.

For detection of cytokines and MPO activity, BALF was centrifuged at 480 X *g* for 5 min at 4°C, and supernatants were recovered for cytokine analysis. The pellets were resuspended in 1 ml NH_4_Cl (0.15 M) and centrifuged at 480 X *g* for 5 min to lyse red blood cells. The supernatants were discarded, and the pellets were resuspended in 0.5% cetyltrimethylammonium chloride (250 μl/mouse) and centrifuged as before. The cleared extracts were used for MPO assay.

### Macrophage cell culture and LPS stimulation

Bone marrow cells were flushed from femurs and tibias of wild-type (+/+) and RCAN1-deficient (-/-) mice. Cells were cultured in DMEM media supplemented with 10% fetal bovine serum, 1% penicillin/streptomycin and 30% L929 supernatant. Media were changed every 2–3 days by replacing half of the initial volume. After 7 days, cells were treated with 200 ng/ml *P*. *aeruginosa* LPS for various time points or left untreated. After macrophage and *P*. *aeruginosa* LPS co-incubation, cell-free supernatants were collected for measuring cytokine and chemokine production. Cell pellets were used for determining RNA transcript, protein expression, and transcription factor activation levels.

### BALF alveolar macrophage collection and LPS stimulation

Alveolar macrophage collection from BALF was described previously [22]. Briefly, BALF was obtained by lavage of lungs with 1 ml of PBS for 3 times. BALF cells were spun down and resuspended in DMEM media containing 10% FBS and 1% penicillin/streptomycin. Cells were incubated at 37 °C for 1 h, which allowed alveolar macrophages to adhere to the plate; poorly attached and unattached cells were removed by washing with PBS. The purity of alveolar macrophage preparations was examined using a Diff-Quik stain set (Siemens Healthcare Diagnostics, DE). Subsequently, wild-type and RCAN1-deficient alveolar macrophages were stimulated with 200 ng/ml *P*. *aeruginosa* LPS for 6 h or left untreated, and supernatants were subjected to ELISA for determining cytokine and chemokine production.

### Cytokine production

Concentrations of IL-6, TNF, MIP-2, RANTES and IP-10 in lungs, BALF and culture supernatants were determined by enzyme-linked immunosorbent assay (ELISA) as described previously using antibody pairs from R&D Systems (Minneapolis, MN) [23]. IFNβ levels were measured using VeriKine-HS Mouse IFNβ ELISA Kits (PBL Assay Science, Piscataway, NJ) according to the manufacturer’s instructions.

### Myeloperoxidase (MPO) assay

The MPO assay was used to determine the infiltration of neutrophils into the lungs of the mice as described previously [24]. Briefly, samples in duplicate (75 μl) were mixed with equal volumes of the substrate (3,3’,5,5’-tetramethyl-benzidine dihydrochloride, 3 mM; Resorcinol, 120 μM; and H_2_O_2_, 2.2 mM) for 2 minutes. The reaction was stopped by adding 150 μl of 2 M H_2_SO_4_. The optical density was measured at 450 nm.

### Western blotting

Cells were lysed in radioimmunoprecipitation assay buffer supplemented with a mixture of protease and phosphatase inhibitors. Cleared lysates (30 μg protein) were electrophoresed in 10% SDS polyacrylamide gels. Gels were transferred to polyvinylidene difluoride membrane, blocked with 5% nonfat milk powder, probed with primary and secondary antibodies, and detected by an ECL-detection system (Western Lightning Plus-ECL; PerkinElmer) on BioMax film (Kodak). Blots were scanned and quantified using ImageJ software.

### Real-time quantitative PCR

Cells were processed using Trizol (Invitrogen) and purified using RNeasy kit (Qiagen). The total RNA was reverse transcribed into cDNA using Reverse Transcriptase (Clontech). *RCAN1-1* primer sequences, Forward 5'- GTTCGTGGACGGCCTGTG -3' and Reverse 5'- AAGGGGTTGCTGAAGTTTATCC -3'. *RCAN1-4* primer sequences, Forward 5'- TGCTTGTGTGGCAAACGATG -3' and Reverse 5'- AGGAACTCGGTCTTGTGCAG -3'. *IL-6* primer sequences, Forward 5'- TAGTCCTTCCTACCCCAATTTCC -3' and Reverse 5'- TTGGTCCTTAGCCACTCCTTC -3'. *TNF* primer sequences, Forward 5'- CATCTTCTCAAAATTCGAGTGACAA -3' and Reverse 5'- TGGGAGTAGACAAGGTACAACCC -3'. *MIP2* primer sequences, Forward 5'- CCACTCTCAAGGGCGGTCAA -3' and Reverse 5'- GGTACGATCCAGGCTTCCCG -3'. *IFNβ* primer sequences, Forward 5'- GCCTTTGCCATCCAAGAGATGC -3' and Reverse 5'- ACACTGTCTGCTGGTGGAGTTC -3'. *RANTES* primer sequences, Forward 5'- CCTGCTGCTTTGCCTACCTCTC -3' and Reverse 5'- ACACACTTGGCGGTTCCTTCGA -3'. *IP-10* primer sequences, Forward 5'- ATCATCCCTGCGAGCCTATCCT -3' and Reverse 5'- GACCTTTTTTGGCTAAACGCTTTC -3'. *IRF3* primer sequences, Forward 5'- CGGAAAGAAGTGTTGCGGTTAGC -3' and Reverse 5'-CAGGCTGCTTTTGCCATTGGTG -3'. *IRF7* primer sequences, Forward 5'- ACAGGGCGTTTTATCTTGCG -3' and Reverse 5'- TCCAAGCTCCCGGCTAAG- 3'. Primers were designed by Primer-BLAST (NCBI). According to manufacturer’s instruction, q-PCR arrays were conducted in triplicate and the mRNA levels were quantified using SYBR Green method on a sequence detection system (ABI Prism 7000; Applied Biosystems). Hypoxanthine-guanine phosphoribosyltransferase (HPRT) was used as a housekeeping control mRNA. Data were analyzed using relative standard curve method according to the manufacturer’s protocol.

### Nuclear extract preparation and electrophoresis mobility shift assay (EMSA)

An electrophoretic mobility shift assay (EMSA) was performed as previously described [25]. Briefly, nuclear protein extracts were prepared using a nuclear extract kit (Active Motif, Carlsbad, CA), following the manufacturer’s protocol. Probe labeling was accomplished by treatment with T4 kinase (Life Technologies, Burlington, ON, Canada) in the presence of ^32^P adenosine triphosphate (Perkin Elmer, Waltham, MA). Labeled oligonucleotides were purified on a Sephadex G-25M column (GE Healthcare, Pittsburgh, PA). Nuclear protein (10 μg) was added to a 10 μl volume of binding buffer supplemented with 1 μg poly(dI:dC) (GE Healthcare) for 15 minutes at room temperature. Labeled double-stranded oligonucleotide was added to each reaction mixture that was incubated at room temperature for 30 minutes and separated by electrophoresis on a 6% polyacrylamide gel in 0.5 X Tris-boric acid-EDTA buffer. Gels were vacuum-dried and subjected to autoradiography. The following synthesized double-stranded oligonucleotides were used: ISRE-binding consensus sequence on mouse IFN-β promoter, 5’-GAAAACTGAAAGGGAGAACTGAAA-3’ [26]; and NF-κB consensus sequence on the IL-6 promoter, 5’-AGTTGAGGGGACTTTCCCAGGC-3’ (Promega, Madison, WI).

Supershift assay was performed as described previously [27]. Briefly, samples were prepared as described above and then incubated with 2 μg of the indicated antibody on ice for 45 minutes prior to incubation with ^32^P-labeled double-stranded DNA probes. Samples were resolved and developed as described above. The antibodies for IRF3 (sc-9082x) and IRF7 (sc-9083x) from Santa Cruz Biotechnology were applied in supershift assay.

### Measurement of IRF7 activation by ELISA

IRF7 activity in cell nuclear extracts was determined using transcription factor ELISA (TransAM IRF7 kit, Active Motif, Carlsbad, CA), according to the manufacturer’s instruction. Briefly, nuclear extracts were added into a 96-well plate pre-coated with oligonucleotides containing the IRF7 consensus binding sites, followed by sequential incubations with IRF7 antibody and HRP-labeled secondary antibody. Results were read on a spectrophotometer at 450 nm.

### Statistical analysis

Data are presented as means ± SEM of the indicated number of experiments. Statistical significance between multiple treatments was determined by one-way analysis of variance and post hoc Tukey’s honest significance test. Alternatively, when two independent variables were analyzed, a two-way analysis of variance and a Bonferroni multiple-comparison test were used. Statistical analysis was performed using GraphPad Prism software version 5.04 (GraphPad Software Inc., La Jolla, CA).

## Results

### RCAN1 deficiency upregulates MyD88-NF-κB-mediated cytokine and chemokine production but downregulates TRIF-IRF-ISRE-mediated cytokine and chemokine production in macrophages following *P*. *aeruginosa* LPS stimulation

**Fig 1:**
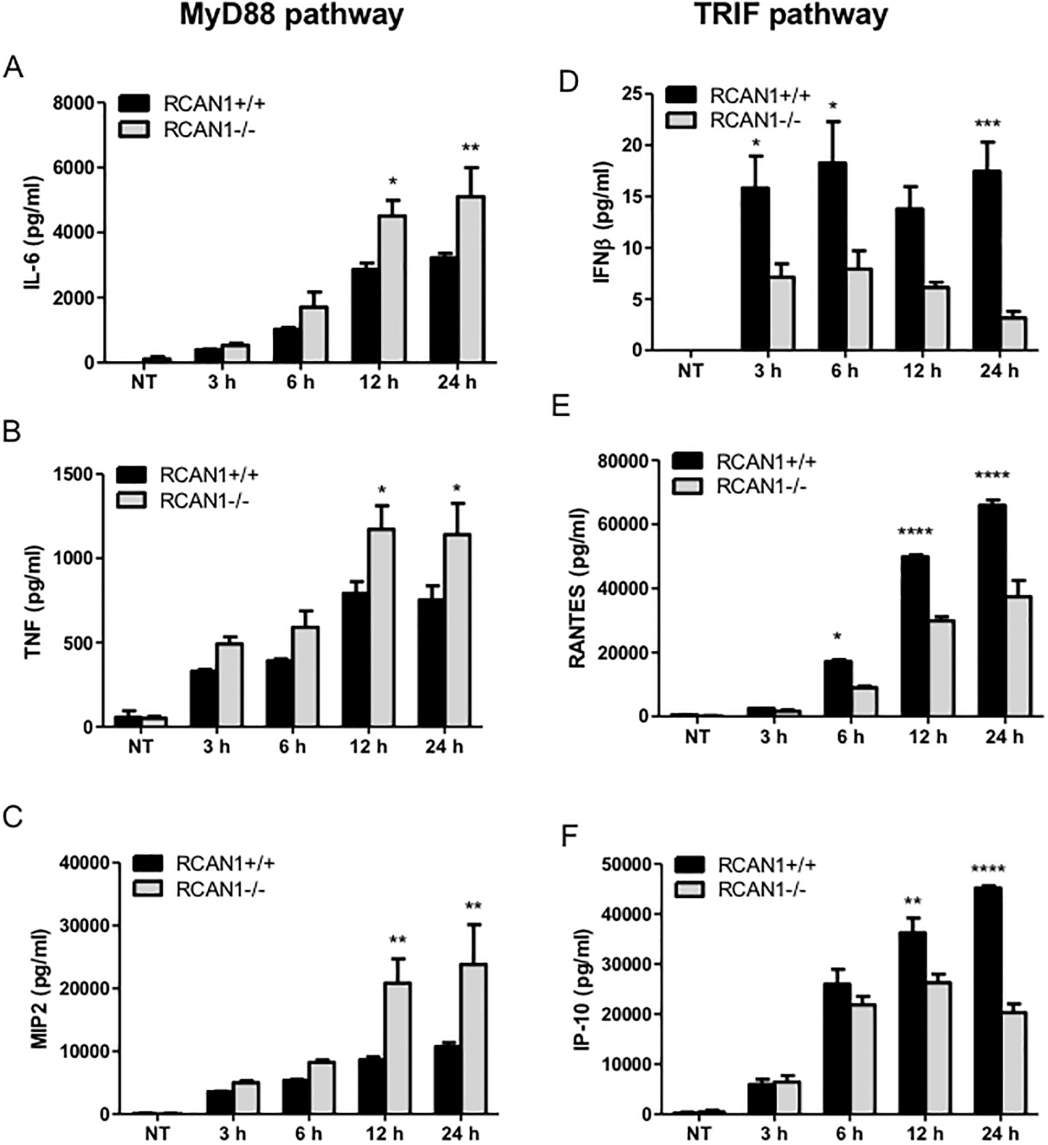
RCAN1 deficiency upregulates MyD88-NF-κB-mediated cytokine and chemokine production but downregulates TRIF-IRF-ISRE-mediated cytokine and chemokine production in BMMs during *P*. *aeruginosa* LPS stimulation. Wild-type (+/+) and RCAN1-deficient (-/-) BMMs were stimulated with 200 ng/ml *P*. *aeruginosa* LPS for 3 h, 6 h, 12 h, 24 h or left untreated (NT). Cell supernatants were collected for the determination of IL-6 (A), TNF (B), MIP2 (C), IFNβ (D), RANTES (E) and IP-10 (F) secretion by ELISA (n = 3 ± SEM, *p<0.05, **p<0.01, ***p<0.001 ****p<0.0001).

Macrophages play an important role in host defense and mediation of inflammatory responses, and they express a variety of TLRs to detect invading microbial pathogens [28, 29]. Treatment of macrophages with *P*. *aeruginosa* LPS led to a significant induction of RCAN1-4 mRNA at 2 h. By contrast, RCAN1-1 mRNA expression was not induced by *P*. *aeruginosa* LPS. This finding suggests that the upregulated RCAN1-4 may be involved in regulation of TLR signaling (S1 Fig). Deletion of exons 5 and 6 from mouse *RCAN1* gene leads to deficiency of all *RCAN1* products. To assess the effect of RCAN1 on the cytokine production regulated through the MyD88-NF-κB pathway, we stimulated wild-type and RCAN1-deficient bone marrow-derived macrophages (BMMs) with 200 ng/ml of *P*. *aeruginosa* LPS for 3 h, 6 h, 12 h, 24 h. Cell supernatants were collected to detect the production of cytokines and chemokines including IL-6, TNF and MIP-2, which are largely regulated through MyD88-NF-κB pathway during *P*. *aeruginosa* infection [30]. We found that the *P*. *aeruginosa* LPS-induced production of IL-6 (Fig 1A), TNF (Fig 1B) and MIP-2 (Fig 1C) was significantly enhanced in RCAN1-deficient BMMs compared to wild-type BMMs, suggesting that RCAN1 negatively regulates MyD88-NF-κB-mediated cytokine and chemokine production. To confirm this finding, the *P*. *aeruginosa* LPS-induced mRNA levels of IL-6, TNF and MIP-2 in wild-type and RCAN1-deficient BMMs were examined by RT-qPCR. We discovered that RCAN1-deficient BMMs displayed elevated mRNA expression of IL-6 (Panel A in S2 Fig), TNF (Panel B in S2 Fig) and MIP-2 (Panel C in S2 Fig) compared to wild-type BMMs in response to *P*. *aeruginosa* LPS stimulation.

IRF3 and IRF7 are transcription factors that translocate to the nucleus upon activation of the TRIF-dependent pathway, and stimulate transcription of IFNβ, RANTES and IP-10, as well as other cytokine genes [31]. The supernatants from wild-type and RCAN1-deficient BMMs challenged with *P*. *aeruginosa* LPS were collected to measure the production of IFNβ, RANTES and IP-10. In contrast to IL-6, TNF and MIP-2, RCAN1-deficient BMMs displayed impaired production of IFNβ (Fig 1D), RANTES (Fig 1E) and IP-10 (Fig 1F), suggesting a positive role of RCAN1 in the regulation of these cytokines and chemokines *in vitro*. Furthermore, the levels of *P*. *aeruginosa* LPS-induced IFNβ (Panel D in S2 Fig), RANTES (Panel E in S2 Fig) and IP-10 (Panel F in S2 Fig) mRNAs were significantly reduced in RCAN1-deficient BMMs compared to wild-type BMMs.

To examine how lung-resident macrophages respond to *P*. *aeruginosa* LPS, the alveolar macrophages were collected from the BALF of wild-type and RCAN1-deficient mice and stimulated with 200 ng/ml of *P*. *aeruginosa* LPS for 6 h or left untreated. Cell supernatants were collected for determining the cytokine and chemokine production. We discovered that the cytokine production pattern of alveolar macrophages was similar to BMMs except IL-6 (S3 Fig).

### RCAN1 deficiency leads to increased *P*. *aeruginosa* LPS-induced IκBα phosphorylation *in vitro*

**Fig 2:**
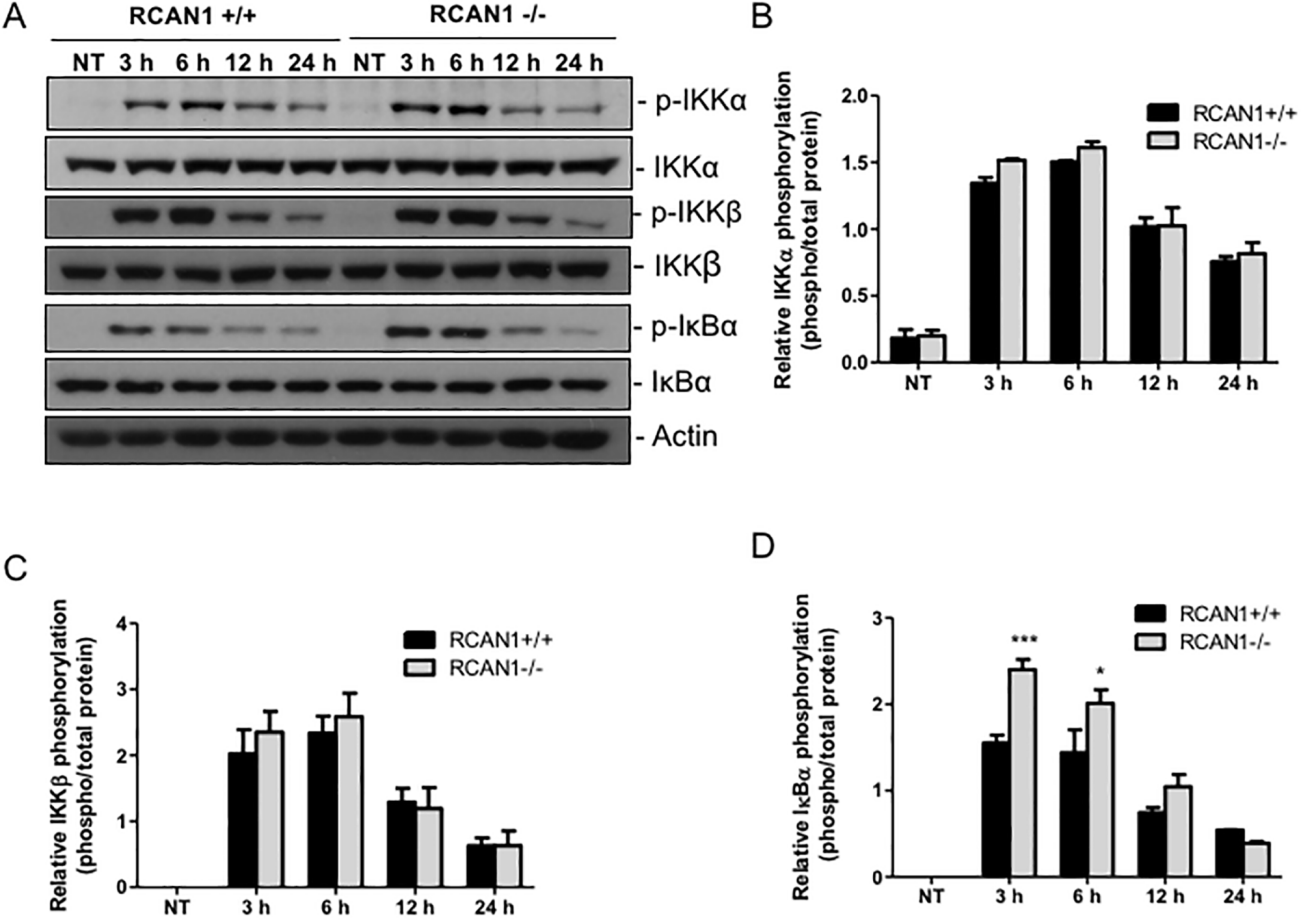
RCAN1 deficiency elevates IκBα phosphorylation *in vitro* following *P*. *aeruginosa* LPS challenge. Wild-type (+/+) and RCAN1-deficient (-/-) BMMs were stimulated with 200 ng/ml *P*. *aeruginosa* LPS for 3 h, 6 h, 12 h and 24 h or left untreated (NT). Cell lysates were subjected to Western blot analysis for phospho- and total IKKα, IKKβ and IκBα, as well as actin as a loading control. Blots are representative of three independent experiments (A). Densitometry analysis of phosphorylated IKKα (B), IKKβ (C) and IκBα (D) was normalized to their total protein respectively (n = 3 ± SEM, *p<0.05, ***p<0.001).

In canonical MyD88-NF-κB signal transduction, the tripartite IKK complex liberates the NF-κB transcription factor by phosphorylating IκBα, thereby stimulating IκBα poly-ubiquitination and degradation by the 26S proteasome; following IκBα degradation, NF-κB translocates to the nucleus where it transactivates genes that regulate immunity, inflammation and cell fate [32]. To understand the molecular mechanisms of RCAN1 regulation of the MyD88-dependent pathway, we characterized the phosphorylation levels of IKK complex subunits IKKα and IKKβ, as well as IκBα, in wild-type and RCAN1-deficient BMMs following *P*. *aeruginosa* LPS challenge at various time points (3 h, 6 h, 12 h and 24 h) by Western blotting (Fig 2A). The *P*. *aeruginosa* LPS-induced phosphorylation of IκBα was markedly enhanced in RCAN1-deficient BMMs compared to wild-type BMMs, whereas no significant differences of IKKα and β phosphorylation levels were observed between wild-type and RCAN1-deficient BMMs (Fig 2B, 2C and 2D), suggesting that IκBα is a potential target site of RCAN1 in *P*. *aeruginosa* LPS-induced MyD88 pathway.

### RCAN1-deficient BMMs display enhanced NF-κB activity in response to *P*. *aeruginosa* LPS stimulation

**Fig 3:**
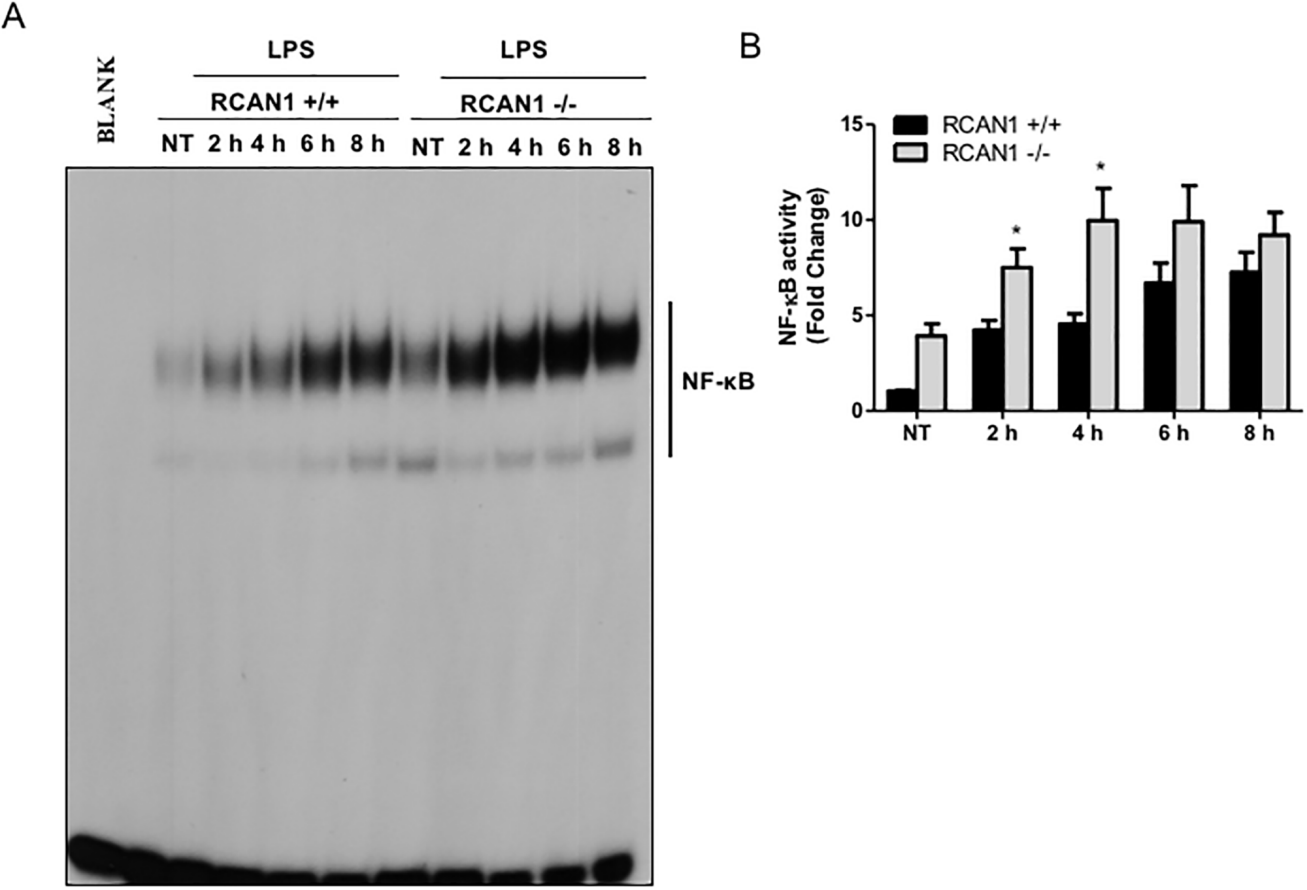
RCAN1 deficiency enhances NF-κB activity *in vitro* following *P*. *aeruginosa* LPS challenge of mouse BMMs. Wild-type (+/+) and RCAN1-deficient (-/-) BMMs were treated with 200 ng/ml *P*. *aeruginosa* LPS for 2 h, 4 h, 6 h, 8 h or left untreated (NT). Nuclear proteins were extracted and subjected to EMSA by incubation with ^32^P-labeled NF-κB DNA probe (A). Data are representative of three individual experiments. Scan densitometry was performed for analysis of NF-κB activity (B), and data are expressed as fold change (n = 3 ± SEM, *p<0.05).

The transcription factor NF-κB is a master regulator of inflammatory responses [33]. To determine whether RCAN1 deficiency has an impact on NF-κB activation *in vitro*, nuclear extracts from *P*. *aeruginosa* LPS-challenged or untreated wild-type and RCAN1-deficient BMMs were subjected to EMSA to analyze NF-κB activity. NF-κB activity was greatly enhanced in RCAN1-deficient BMMs compared to wild-type BMMs (Fig 3). This finding corroborates our observation of increased IκBα phosphorylation in RCAN1-deficient BMMs, and suggests that RCAN1 negatively regulates the *P*. *aeruginosa* LPS-induced NF-κB activity *in vitro*.

### RCAN1 deficiency impairs TRIF-IRF-ISRE pathway activation in macrophages in response to *P*. *aeruginosa* LPS stimulation

**Fig 4:**
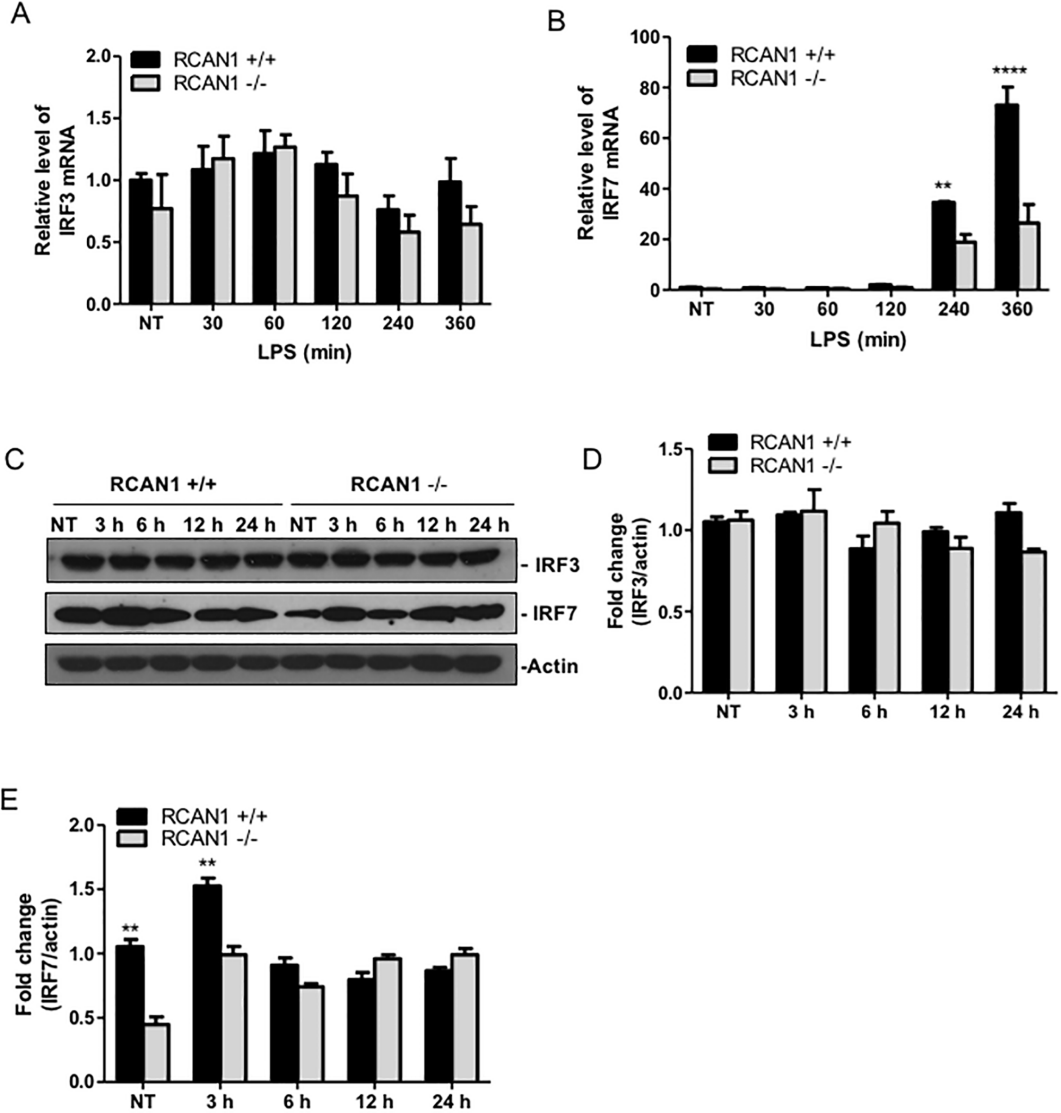
RCAN1-deficient BMMs display diminished IRF7 mRNA and protein expression. Wild type (+/+) and RCAN1-deficient (-/-) BMMs were treated with 200 ng/ml *P*. *aeruginosa* LPS for various time points or left untreated (NT). The total RNA isolated from these cells was reverse transcribed to cDNA and subjected to real-time quantitative PCR for *IRF3* (A), and *IRF7* (B) gene expression. The gene expression was normalized to housekeeping control gene *HPRT*. Cell lysates were immunoblotted to measure IRF3, IRF7 and actin protein levels. Immunoblots are representative of three independent experiments (C). Densitometry analysis of IRF3 and IRF7 levels was normalized to actin (D, E), and data are presented as fold change (n = 3 ± SEM, **p<0.01, ****p<0.0001).

To further demonstrate the regulatory role of RCAN1 in TRIF-IRF-ISRE pathway, we analyzed the *P*. *aeruginosa* LPS-induced mRNA and protein expression of IRF3 and IRF7 in wild-type and RCAN1-deficient BMMs by RT-qPCR and Western blot respectively. There were no significant differences observed in the *P*. *aeruginosa* LPS-induced IRF3 mRNA levels between wild-type and RCAN1-deficient BMMs (Fig 4A). By contrast, RCAN1-deficient BMMs displayed diminished IRF7 mRNA levels compared to the wild-type BMMs following *P*. *aeruginosa* LPS challenge (Fig 4B). Similarly, the IRF3 protein was found to be constitutively expressed during LPS treatment, whereas the IRF7 protein levels were significantly elevated at 3 h and RCAN1-deficient BMMs displayed reduced IRF7 protein expression compared to wild-type BMMs (Fig 4C, 4D and 4E). These results suggest that RCAN1 facilitates IRF7 expression *in vitro*.

**Fig 5:**
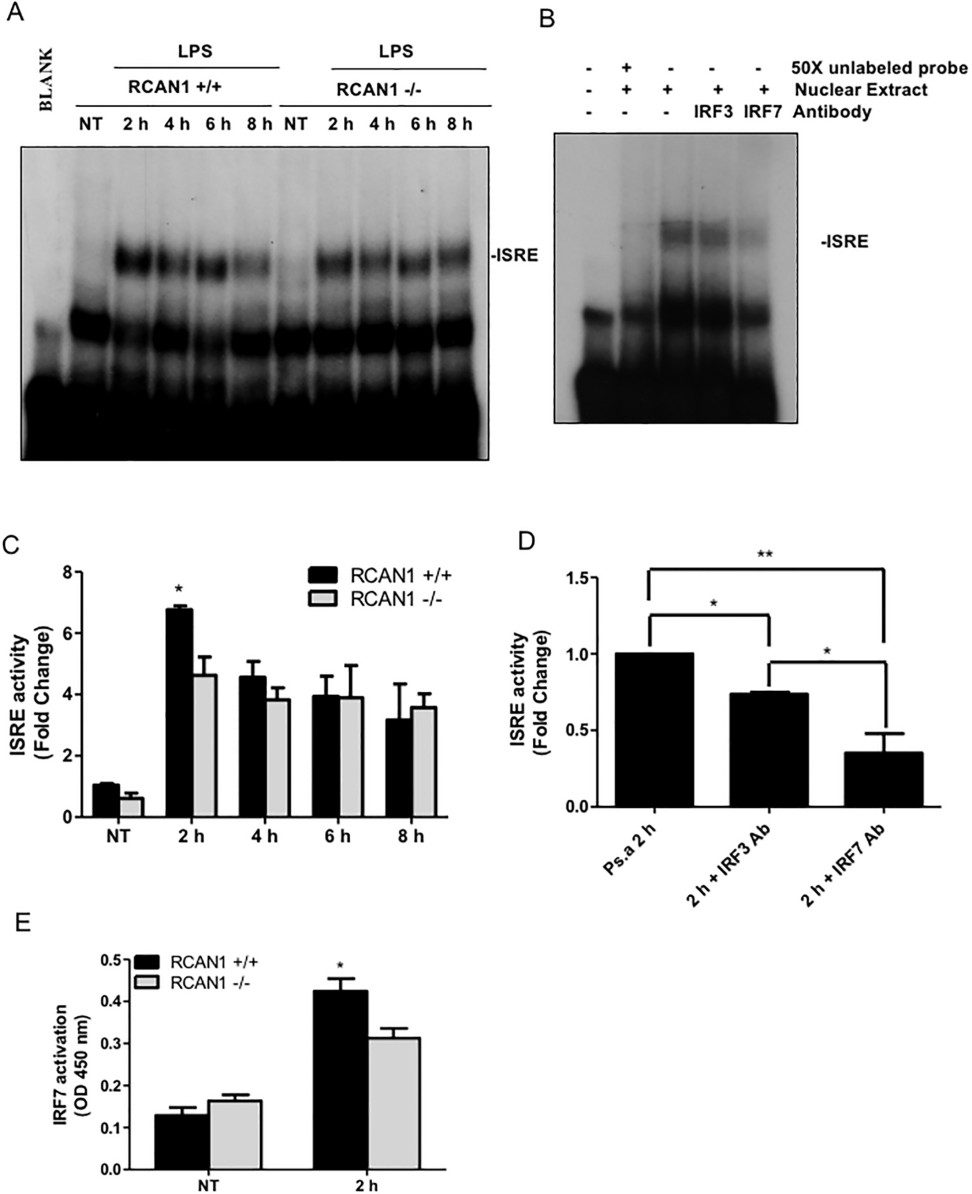
RCAN1 deficiency impairs TRIF-IRF-ISRE activity *in vitro* during *P*. *aeruginosa* LPS stimulation. Wild-type (+/+) and RCAN1-deficient (-/-) BMMs were treated with 200 ng/ml *P*. *aeruginosa* LPS for 2 h, 4 h, 6 h, 8 h or left untreated (NT). Nuclear proteins were extracted and subjected to EMSA by incubation with ^32^P-labeled ISRE DNA probe (A). Data are representative of three individual experiments. Nuclear extracts from wild-type (+/+) BMMs treated with 200 ng/ml *P*. *aeruginosa* LPS for 2 h were incubated with or without specific antibodies to IRF3 and IRF7 for 1 h or 50 X unlabeled ISRE probe for 30 min at room temperature before EMSA experiment using the ^32^P-labeled ISRE probe (B). Data are representative of three individual experiments. Scan densitometry was performed for analysis of ISRE activity (C, D), and data are expressed as fold change. Cell nuclear extracts from NT and LPS 2 h stimulated wild-type and RCAN1-deficient BMMs were subjected to transcription factor ELISA for determining IRF7 activity (E). (n = 3 ± SEM *p<0.05, **p<0.01).

After nuclear translocation, IRF3 and IRF7 induce transcription through binding to ISRE sites in the promoters of target genes [6]. Using EMSA, we tested the *P*. *aeruginosa* LPS-induced ISRE binding activity in wild-type and RCAN1-deficient BMMs. Wild-type and RCAN1-deficient BMMs were treated with 200 ng/ml *P*. *aeruginosa* LPS for 2, 4, 6 and 8 h or left untreated. ISRE binding activity was significantly reduced but not abolished in the RCAN1-deficient BMMs at 2 h, compared to wild-type BMMs (Fig 5A and 5C). Because both IRF3 and IRF7 can contribute to ISRE binding activity, supershift assays for IRF3 and IRF7 were performed on nuclear extracts from 2 h LPS-stimulated wild-type BMMs, using IRF3 and IRF7 antibodies to determine whether the two proteins contribute to ISRE binding. The IRF3 and IRF7 antibodies employed in these supershift assays were previously validated by us and others [34, 35]. Both IRF3 and IRF7 antibodies blocked ISRE binding activity, and the IRF7 antibody resulted in a greater reduction of ISRE binding activity than the IRF3 antibody (Fig 5B and 5D). Moreover, the binding specificity of nuclear proteins to ISRE DNA sequence was verified through competitive binding by 50 X non-radioisotope labeled ISRE probes (Fig 5B). These findings suggest that RCAN1 contributes to ISRE binding activity and IRF7 is predominant in binding of ISRE in response to *P*. *aeruginosa* LPS stimulation. To further demonstrate RCAN1-regulated IRF7 activation, the nuclear extracts from NT and 2 h LPS-stimulated wild-type and RCAN1-deficient BMMs were examined by transcription factor ELISA for IRF7. Consistent with the EMSA results, reduced IRF7 activation at 2 h was observed in RCAN1-deficient BMMs compared with wild-type BMMs (Fig 5E).

### RCAN1 differentially regulates the activation of mitogen-activated protein kinases

TLR signaling is able to activate mitogen-activated protein kinases (MAPKs), including p38, ERK, and JNK, which are important for mediation of inflammatory gene expression [2]. To determine whether RCAN1 plays a role on MAPK activation, cell lysates from *P*. *aeruginosa* LPS-activated wild-type and RCAN1-deficient BMMs were subjected to Western blotting to assess the phosphorylation levels of ERK, JNK, and p38 (S4 Fig). Interestingly, RCAN1-deficient BMMs displayed significantly increased phosphorylation of ERK but reduced phosphorylation of JNK compared to wild-type BMMs (Panel B and C in S4 Fig). Moreover, no statistically significant differences in p38 phosphorylation were observed between wild-type and RCAN1-deficient BMMs (Panel D in S4 Fig). These findings suggest that RCAN1 differentially regulates MAPK signaling pathways in our system.

### RCAN1-deficient mice display enhanced MyD88-NF-κB-mediated cytokine and chemokine production *in vivo* during *P*. *aeruginosa* LPS stimulation

**Fig 6:**
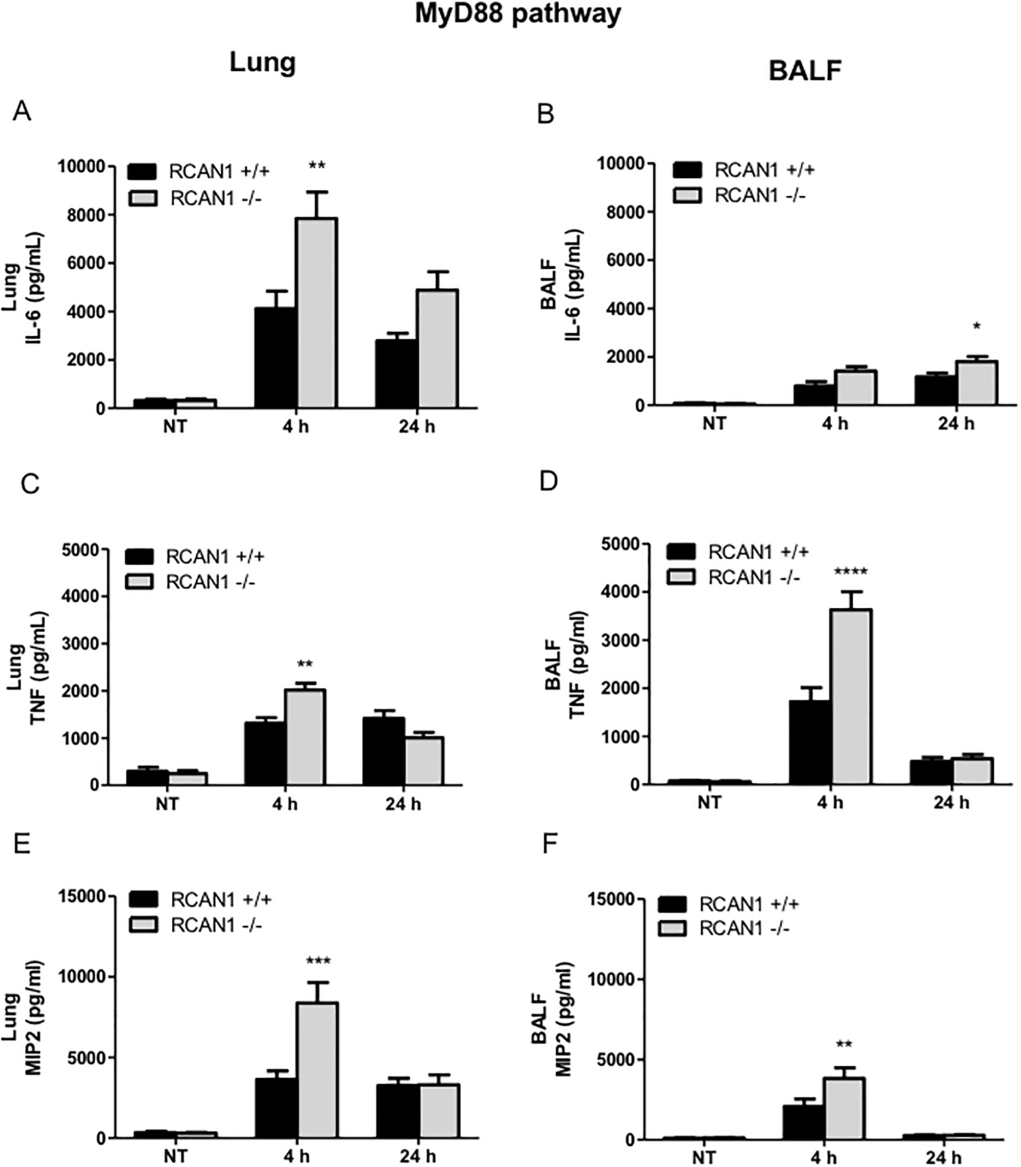
RCAN1-deficient mice display enhanced MyD88 pathway-mediated proinflammatory cytokine and chemokine production in response to *P*. *aeruginosa* LPS stimulation *in vivo*. Wild-type (+/+) and RCAN1-deficient (-/-) mice were treated intranasally with 1 μg *P*. *aeruginosa* LPS per gram of body weight, or an equivalent volume of saline as a control (NT) for 4 h or 24 h. After 4 h or 24 h, lung tissues and BALF were collected for determination of IL-6 (A, B) and TNF (C, D), MIP2 (E, F) production by ELISA. (n = 9 ± SEM, *p<0.05, **p<0.01, ***p<0.001, ****p<0.0001).

To investigate the role of RCAN1 in TLR-MyD88-dependent pathway *in vivo*, we incorporated RCAN1-deficient mice into a model of *P*. *aeruginosa* LPS-induced acute pneumonia. Wild-type and RCAN1-deficient mice were intranasally administered with 1 μg *P*. *aeruginosa* LPS per gram of body weight for 4 h or 24 h. Lung tissues and BALF were collected to determine the MyD88-NF-κB-mediated production of IL-6 (Fig 6A and 6B), TNF (Fig 6C and 6D) and MIP-2 (Fig 6E and 6F) by ELISA. RCAN1-deficient mice displayed enhanced production of IL-6, TNF and MIP2 in lungs and BALF compared to wild-type mice, which was consistent with the pattern of *in vitro* cytokine production.

**Fig 7:**
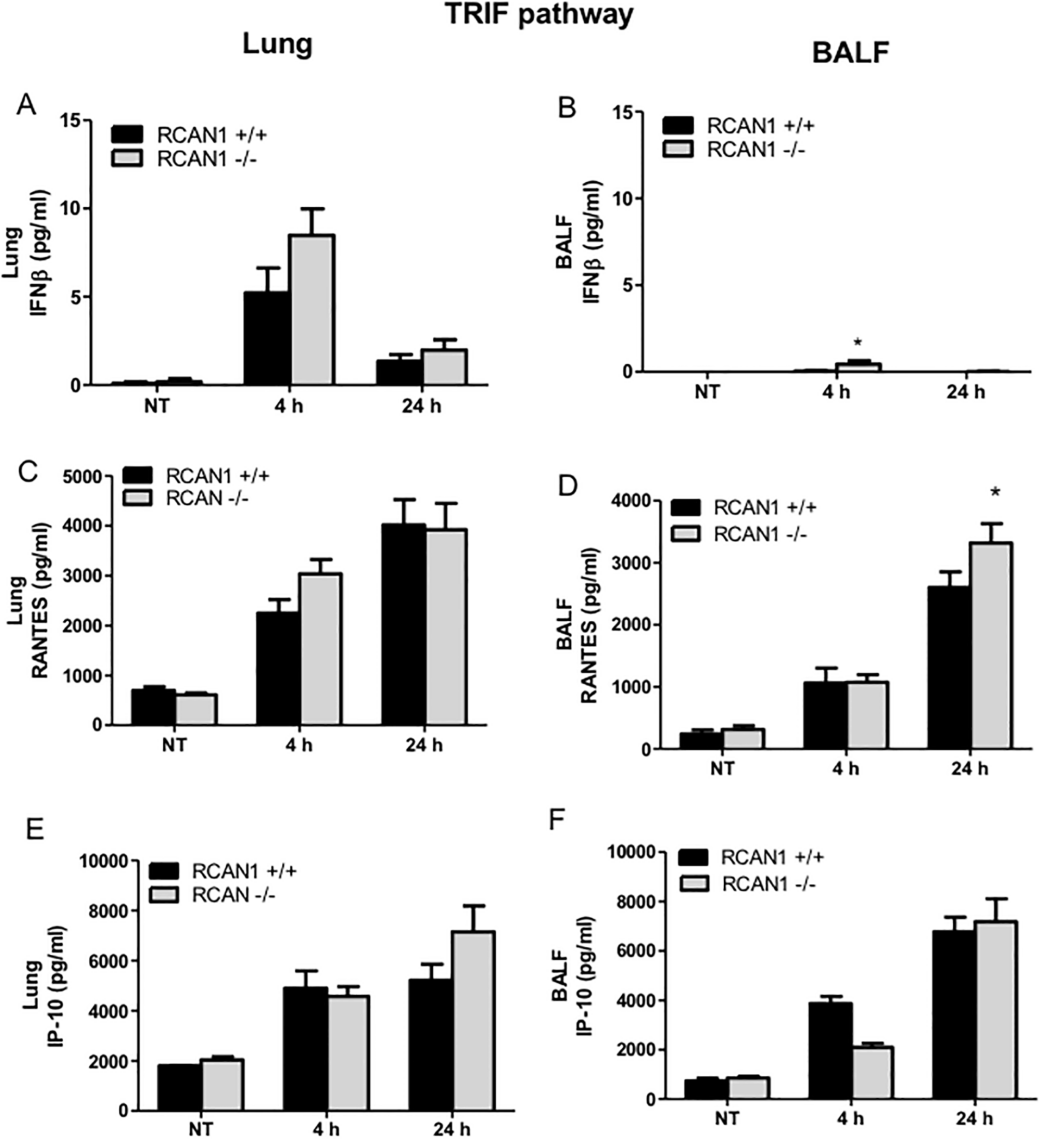
RCAN1 deficiency has minor effects on TRIF-IRF-ISRE-regulated cytokine and chemokine production following *P*. *aeruginosa* LPS stimulation in lung. Wild-type (+/+) and RCAN1-deficient (-/-) mice were administered intranasally with 1 μg *P*. *aeruginosa* LPS per gram of body weight, or an equivalent volume of saline as a control (NT) for 4 h or 24 h. After 4 h or 24 h, lung tissues and BALF were collected for the determination of IFNβ (A, B), RANTES (C, D) and IP-10 (E, F) production by ELISA (n = 9 ± SEM, *p<0.05).

The *in vivo* products of the TRIF-ISRE pathway including IFNβ (Fig 7A and 7B), RANTES (Fig 7C and 7D) and IP-10 (Fig 7E and 7F) were analyzed by ELISA. Compared to the *in vitro* data, RCAN1 deficiency only partially affected these cytokine and chemokine production. In the lungs, there are no significant differences in IFNβ, RANTES, and IP-10 production observed between wild-type and RCAN1-deficient mice following *P*. *aeruginosa* LPS administration. In the BALF, there is an increased production of IFNβ at 4 h and RANTES at 24 h in RCAN1-deficient mice, and no significant difference in IP-10 between wild-type and RCAN1-deficient mice.

### RCAN1-deficient mice display increased NF-κB activity and reduced ISRE binding activity *in vivo* following *P*. *aeruginosa* LPS challenge

**Fig 8:**
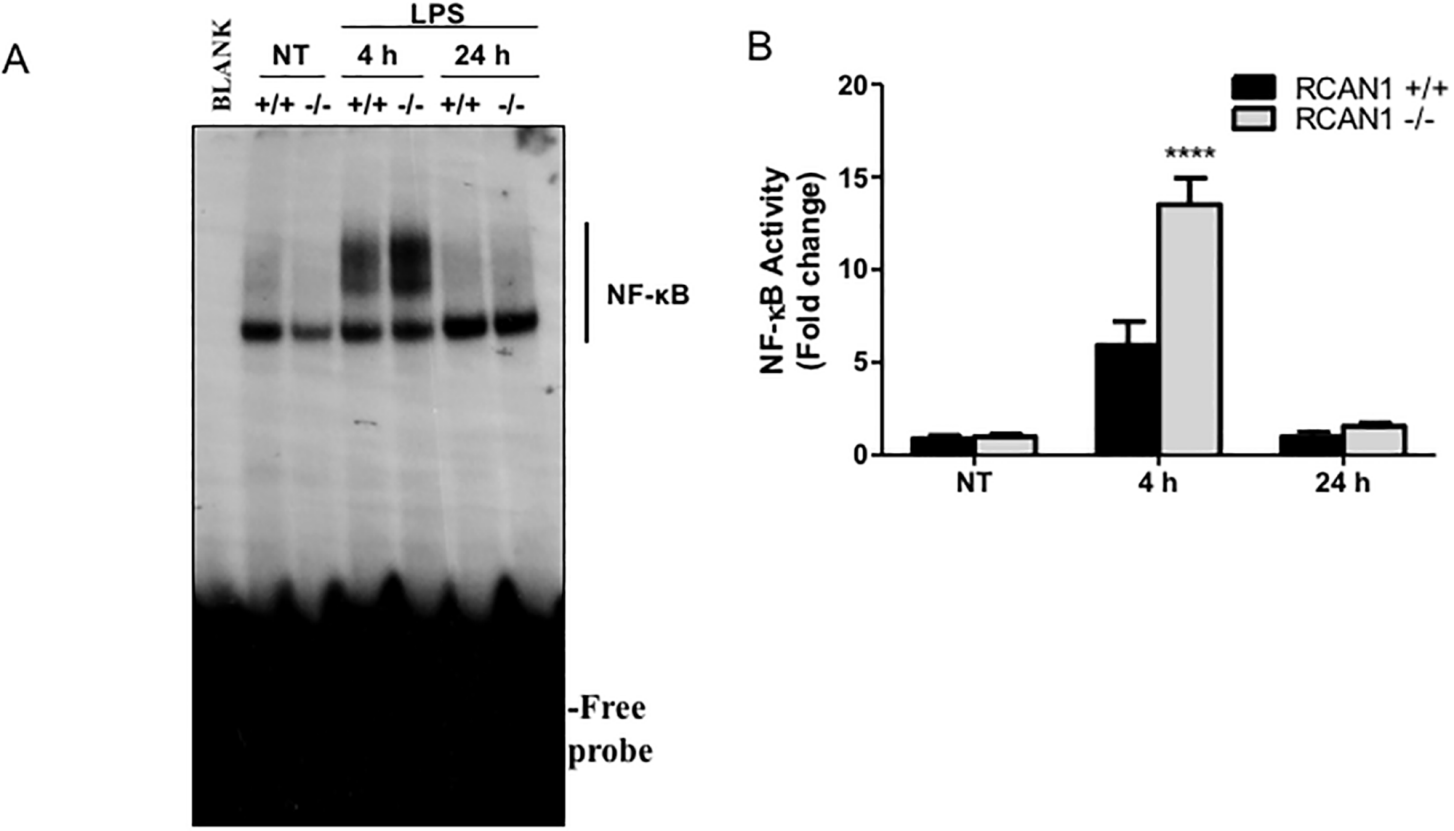
RCAN1-deficicent mice show increased activity of transcription factor NF-κB *in vivo* following *P*. *aeruginosa* LPS challenge. Wild-type (+/+) and RCAN1-deficient (-/-) mice were challenged intranasally with 1 μg *P*. *aeruginosa* LPS per gram of body weight, or an equivalent volume of saline as a control (NT) for 4 h or 24 h. Nuclear proteins were extracted from lung tissues and subjected to EMSA by incubation with ^32^P-labeled NF-κB DNA probe (A). Data are representative of six individual experiments. Scan densitometry was performed for analysis of NF-κB activation (B), and data are expressed as fold change versus wild-type untreated lungs (n = 6 ± SEM, ****p<0.0001).

**Fig 9:**
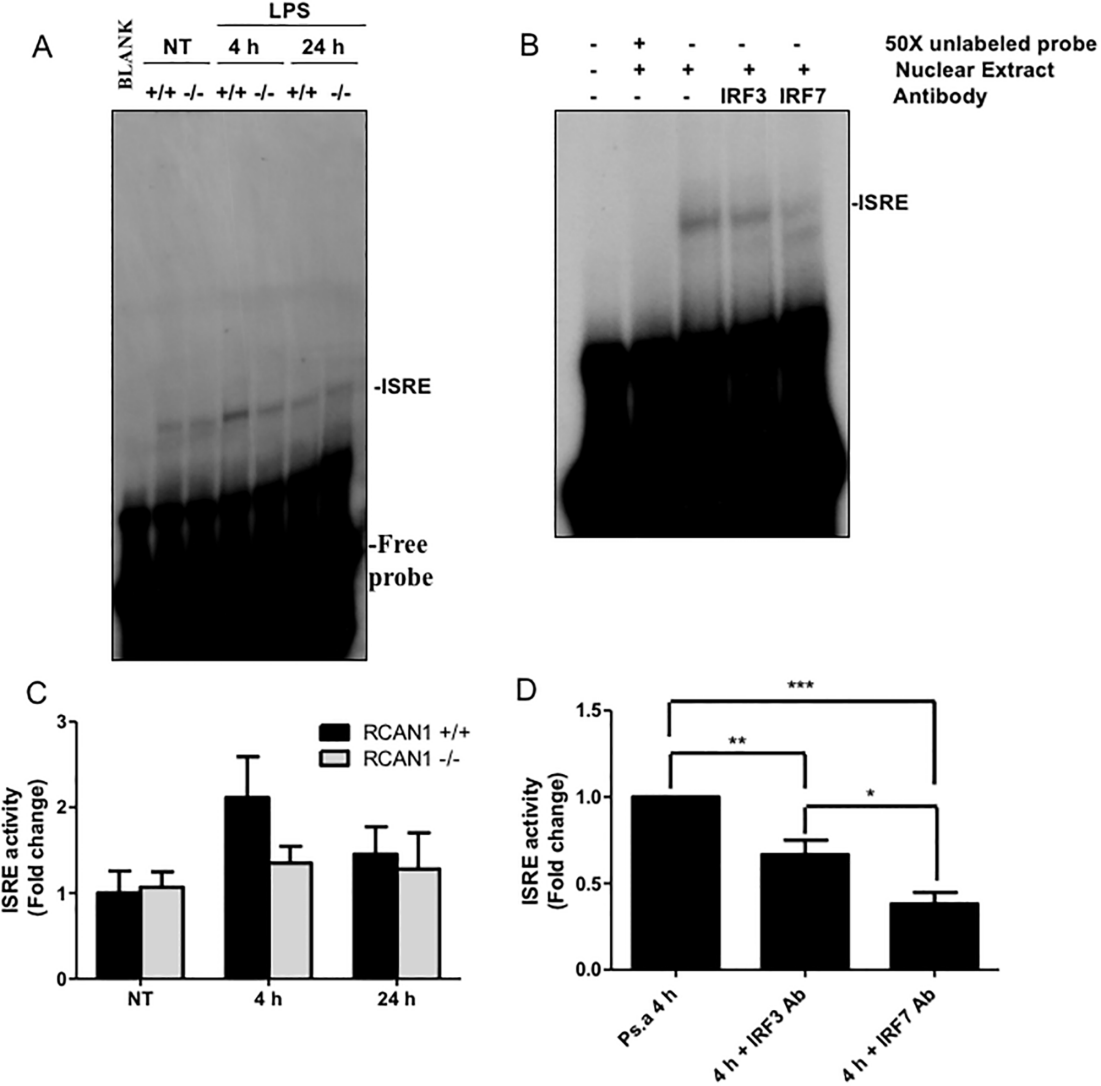
RCAN1 deficiency does not significantly affect TRIF-IRF-ISRE activity in lung following *P*. *aeruginosa* LPS challenge. Wild-type (+/+) and RCAN1-deficient (-/-) mice were challenged intranasally with 1 μg *P*. *aeruginosa* LPS per gram of body weight, or an equivalent volume of saline as a control (NT) for 4 h or 24 h. Nuclear proteins were extracted from lung tissues and subjected to EMSA by incubation with ^32^P-labeled ISRE DNA probe (A). Data are representative of six individual experiments. Nuclear proteins from the lungs of wild-type (+/+) mice treated with *P*. *aeruginosa* LPS for 4 h were incubated with or without specific antibodies to IRF3 and IRF7 for 1 h or 50 X unlabeled ISRE probe for 30 min at room temperature before EMSA experiment using the ^32^P-labeled ISRE probe (B). Data are representative of five individual experiments. Scan densitometry was performed for analysis of ISRE activity (C, D), and data are expressed as fold change. n = 6 ± SEM (C). n = 5 ± SEM *p<0.05, **p<0.01, ***p<0.001 (D).

To determine the TLR-mediated activities of transcription factors *in vivo*, nuclear extracts from the lungs of *P*. *aeruginosa*-challenged wild-type and RCAN1-deficient mice were subjected to EMSA for NF-κB and ISRE activity. *P*. *aeruginosa* LPS-induced NF-κB activity was significantly enhanced in the lungs of RCAN1-deficient mice at 4 h compared to wild-type mice (Fig 8A and 8B). By contrast, a trend of decreased ISRE binding activity at 4 h in RCAN1-deficient mice was observed. However, this result failed to reach statistical significance (Fig 9A and 9C). Moreover, we also determined the binding specificity of LPS-induced ISRE to IRF3 and IRF7 by performing a supershift assay. Consistent with *in vitro* results, anti-IRF3 antibody and anti-IRF7 antibody reduced LPS-induced ISRE binding activity (Fig 9B and 9D). Additionally, the IRF3 mRNA expression in the lungs of wild-type and RCAN1-deficient mice was not induced in response to *P*. *aeruginosa* LPS stimulation, whereas a trend of decreased IRF7 mRNA level at 4 h was observed in the lungs of RCAN1-deficient mice compared to the wild-type mice, which did not reach statistical significance (S5 Fig). These results demonstrate that RCAN1 plays a negative regulatory role in NF-κB activity *in vivo* and RCAN1 deficiency has a limited impact on ISRE binding activity *in vivo*.

### RCAN1-deficent mice have increased neutrophil recruitment *in vivo* following *P*. *aeruginosa* LPS stimulation

TLR signaling is essential for recruitment of neutrophils to the site of injury or bacterial infection [36, 37]. To examine the impact of RCAN1 deficiency on neutrophil infiltration *in vivo*, the lung and BALF lysates from LPS-challenged wild-type and RCAN1-deficient mice at 4 h and 24 h were collected to measure the activity of the neutrophil granule-specific enzyme myeloperoxidase (MPO) (Panel A and B in S6 Fig). A significantly increased MPO activity was found in the lungs of RCAN1-deficient mice compared to wild-type mice. In addition, the lung histology data suggest that RCAN1-deficient mice have enhanced neutrophil recruitment in response to *P*. *aeruginosa* LPS stimulation compared to wild-type counterparts (Panel C in S6 Fig). These results indicate that RCAN1 plays an important role in LPS-induced neutrophil recruitment in the lungs.

## Discussion

TLRs are highly conserved pattern-recognition receptors that are essential for production of proinflammatory cytokines and antimicrobial mediators in innate immunity [38]. Ligand binding to TLRs activates two distinct pathways, MyD88-NF-κB and TRIF-IRF-ISRE, which are tightly controlled in healthy individuals [1]. Negative regulation of TLR signaling is essential for maintaining proper homeostasis and preventing immune pathology [15]. Although many negative regulators have been identified in the past decade, the molecular mechanisms of how these negative regulators govern TLR signaling are incompletely understood. We previously identified a small evolutionary conserved protein, RCAN1, as a central negative regulator of inflammation during *P*. *aeruginosa* infection [16]. Herein, we utilized *P*. *aeruginosa* LPS to directly activate TLR4 signaling and revealed a differential role of RCAN1 in regulation of MyD88-NF-κB and the TRIF-IRF-ISRE pathways *in vitro*: RCAN1 downregulates MyD88-NF-κB pathway through inhibition of IκBα phosphorylation, and promotes activation of TRIF-ISRE pathway through regulation of IRF7 activation and expression (S7 Fig). The *in vivo* results support an inhibitory role of RCAN1 in the MyD88-NF-κB pathway, and the impact of RCAN1 deficiency on neutrophil recruitment suggests an important role of RCAN1 in host defense against microbial infection.

The RCAN1 gene consists of 7 exons, of which exons 1–4 can be alternatively spliced into different transcript isoforms [39]. Alternative splicing and differential promoter usage contribute to generation of different RCAN1 isoforms. The two main isoforms, RCAN1-1 and RCAN1-4, contain exons 1, 5, 6, 7 and exons 4, 5, 6, 7, respectively, and have been identified in a variety of tissues. By contrast, RCAN1-2 and RCAN1-3 proteins are not detectable in tissues and their functions are not clear [18]. However, the expression of the isoforms RCAN1-1 and RCAN1-4 is regulated differently. RCAN1-1 is constitutively expressed in most tissues, whereas the transcription of RCAN1-4 is induced by several stimuli, including intracellular Ca^2+^, vascular endothelial growth factor (VEGF), injury and oxidative stress [40–43]. In this study, we found that *P*. *aeruginosa* LPS largely induced mRNA expression of RCAN1-4, but not RCAN1-1 in macrophages, suggesting a potential involvement of RCAN1-4 in LPS-activated TLR4 signaling. Additionally, the inhibitory effects of RCAN1-1 on NF-κB activity have also been previously identified [44]. Thus, it remains possible that the constitutively expressed RCAN1-1 also plays a role in regulation of TLR signaling.

In the MyD88-NF-κB pathway, we discovered an enhanced phosphorylation level of IκBα, but not IKKα and IKKβ, in *P*. *aeruginosa* LPS-stimulated RCAN1-deficient BMMs. This led to increased NF-κB activation and upregulated IL-6, TNF and MIP2 production. Previous studies have shown that RCAN1 is able to inhibit NF-κB activation by affecting IκBα phosphorylation, and the regulation of IκBα by RCAN1 can be achieved through calcineurin-dependent [45–48] or -independent mechanisms [44, 49]. Calcineurin is a Ca^2+/^calmodulin-dependent serine/threonine phosphatase that consists of a catalytic subunit, calcineurin A, and a regulatory subunit, calcineurin B [50]. RCAN1 interacts with calcineurin A and inhibits the calcineurin-dependent phosphatase activity [20]. Calcineurin was previously shown to facilitate NF-κB activation [46–48, 51, 52]. A study reported that calcineurin synergizes with protein kinase C-dependent pathway to enhance NF-κB DNA binding activity by inducing phosphorylation and degradation of IκBα in T-cell lines [47]. Moreover, constitutively active expression of calcineurin in muscle C2C12 cells is associated with increased phosphorylation level of IκBα [48]. Additionally, calcineurin has been identified to upregulate TCR-induced NF-κB activity through interaction with Carma1-Bcl10-Malt1 complex and dephosphorylation of Bcl10 in T-cells [51, 52]. By contrast, RCAN1 has also been found to inhibit NF-κB activation independent of calcineurin activity [44, 49]. A recent study found that the N-terminal domain RCAN1 directly interacts with IκBα and affects the phosphorylation of IκBα at tyrosine 42 in HEK293 cells [44]. Therefore, it is possible that RCAN1 suppresses *P*. *aeruginosa* LPS-induced MyD88-NF-κB pathway indirectly through inhibition of calcineurin or directly interacts with IκBα.

RCAN1 inhibits the NFAT pathway by limiting calcineurin activity [20]. Many studies have demonstrated cooperation between NF-κB and NFAT pathways. NF-κB and NFAT can recognize similar DNA binding sites in target gene promoters, and coordination between them mediates maximal production of cytokines and chemokines [53, 54]. Physical interactions between NF-κB and NFAT have been found in cardiomyocytes, which promote the gene expression for cardiac hypertrophic responses [55]. Moreover, we recently identified that inhibition of NFAT reduced NF-κB DNA binding activity and NF-κB inhibition diminished NFAT DNA binding activity during *P*. *aeruginosa* lung infection [56]. In light of these facts, it is likely that increased NFAT activity contributes to increased *P*. *aeruginosa* LPS-induced NF-κB activation in RCAN1-deficient systems.

There is accumulating evidence for RCAN1’s role in pathway activation; RCAN1 enhances cAMP-induced CREB phosphorylation and CREB-mediated gene transcription in neuronal PC12 cells [57], and mediates neuronal apoptosis by activation of caspase-3 and caspase-9 responsible for apoptotic signaling [58]. Furthermore, the positive role of RCAN1 has previously discovered to depend on post-translational modifications such as phosphorylation and expression level of RCAN1 [59, 60]. Our data provide the first evidence that RCAN1 contributes to the activation of the TRIF-IRF7-ISRE pathway. RCAN1 deficiency impedes *P*. *aeruginosa* LPS-induced ISRE binding activity and TRIF-IRF-ISRE-mediated mRNA and protein expression of IFNβ, RANTES and IP-10 *in vitro*. Impaired IRF7 mRNA and protein expression, but not IRF3, were shown in RCAN1-deficient BMMs, suggesting that RCAN1 facilitates the TRIF-ISRE pathway by targeting IRF7. A study reported that calcineurin negatively regulates the TRIF pathway-mediated IFNβ production in LPS-activated mouse macrophage cell line RAW 264.7 [61]. Thus, RCAN1 may promote TRIF-IRF7-ISRE pathway activation by inhibition of calcineurin. However, considering the ability of RCAN1 to modulate protein phosphorylation, it remains formally possible that RCAN1 directly modulates phosphorylation of IRF7 or related proteins in the pathway.

The TRIF-dependent pathway plays a well-characterized role in host antiviral defense by increasing production of type-I IFN production [62]. Moreover, it also defends against bacterial infection by mediating MyD88-independent activation of NF-kB and production of inflammatory mediators [63]. However, the mechanisms involved in TRIF-mediated host protection against bacterial pathogens are not fully understood. Recent studies have shown that TRIF deficiency reduces production of proinflammatory cytokines and chemokines, such as IFNβ, TNFα, KC, RANTES and IP-10, and diminished neutrophil recruitment, leading to increased bacterial burden and decreased survival [11, 64, 65]. In this study, we dissected the differential roles of the RCAN1 in regulating the production of chemoattractants of neutrophils and how this affected neutrophil recruitment triggered by LPS. We found that the LPS-activated RCAN1-deficient BMMs displayed reduced, but not abolished, RANTES and IP-10 production via the TRIF-IRF-ISRE pathway. By contrast, the MyD88-mediated production of chemokine MIP-2 was greatly enhanced in RCAN1-deficient BMMs. Furthermore, neutrophil recruitment in the lungs of RCAN1-deficient mice was not affected by impaired production of RANTES and IP-10 mediated through TRIF pathway by BMMs. These findings suggest that the role of MyD88-dependent pathway is dominant over TRIF-dependent pathway in bacterial infection. A previous study showed that MyD88-deficient mice manifested a much more remarkable phenotype, including reduced survival and impaired bacterial clearance, compared with TRIF-deficient mice [65]. Thus, our findings support the established model for a dominant role of the MyD88-dependent pathway in response to bacterial infection.

The *in vivo* pattern of TRIF-ISRE-regulated IFNβ, RANTES and IP-10 production in lungs and BALF following *P*. *aeruginosa* LPS stimulation was not consistent with the *in vitro* data. Furthermore, the *in vivo* ISRE binding activity was not significantly impaired in RCAN1-deficient mice, whereas RCAN1 deficiency greatly reduced ISRE binding activity in macrophages. This could be explained by the fact that lung tissues consist of different kinds of immune cells and non-immune cells other than macrophages, and RCAN1 may function differently in these other cell types. Previous reports have described diverse roles of RCAN1 in calcineurin activity, which depends on cell types and cellular context [21, 59, 60, 66, 67]. Phosphorylation of RCAN1 by TAK1 at serine 94 and 136, switches RCAN1 from an inhibitor to a facilitator of calcineurin-NFAT signaling in cardiomyocytes [59]. In cardiac hypertrophic model in mice, RCAN1 promotes calcineurin activity [21]. In contrast, in T cells [67] and many other cell types, RCAN1 inhibits calcineurin activity. Similarly, CpG DNA-induced TLR signaling stimulates IFNβ production in dendritic cells, but not in macrophages [68]. Many cell types have the TRIF pathway capacity including dendritic cells, neutrophils, natural killer (NK) cells, T-cells, lung epithelial cells, lung endothelial cells and fibroblasts in bacterial or viral infection [69–77]. It is possible that these cell types participate in the *in vivo* model of *P*. *aeruginosa* infection. However, how RCAN1 and TRIF function in each one of these cell types is not clear. A combination of the possible inhibitory, stimulatory or no effect of RCAN1 on TLR signaling in different cell types *in vivo* likely contributes to the results that we observed in RCAN1-deficient mice *in vivo*. Although the *in vivo* data in RCAN1-deficient mice could not specifically reveal the role of macrophages, they provide the valuable information of how MyD88-dependent and TRIF-dependent cytokine/chemokine profile changes in RCAN1-deficient animals. Future studies should focus on the investigation of RCAN1 deficiency in macrophages *in vivo*.

In this study, we also identified a differential role of RCAN1 in regulation of MAPK activation, whereby RCAN1 deficiency leads to enhanced ERK phosphorylation and reduced JNK phosphorylation in response to *P*. *aeruginosa* LPS stimulation *in vitro*, supporting the notion that RCAN1 can differentially regulate signal transduction pathways. The enhanced ERK phosphorylation in *P*. *aeruginosa* LPS-stimulated RCAN1-deficient macrophages is consistent with our previous observation of ERK hyperphosphorylation in RCAN1-deficient macrophages during *P*. *aeruginosa* infection [16]. ERK and NF-κB p65 interactions have been previously described [78], and enhanced ERK phosphorylation upregulates NF-κB activity [79–81]. JNK plays a critical role in inducing the expression of pro-apoptotic proteins [82]. Multiple studies have demonstrated crosstalk between NF-κB and JNK activation. NF-κB was found to suppress JNK activation by mediating production of JNK inhibitors [83–85]. In addition, LPS is able to induce apoptosis in macrophages through autocrine secretion of TNFα [86]. Therefore, it is possible that the enhanced NF-κB activation by RCAN1 deficiency would inhibit the *P*. *aeruginosa* LPS-induced JNK phosphorylation and pro-apoptotic events.

Altogether, our findings demonstrate a novel regulatory mechanism of RCAN1 in TLR signaling, which differentially regulates MyD88-NF-κB and TRIF-IRF7-ISRE signaling pathways. This study broadens our understanding of regulation of TLR signaling in innate immunity and suggests that RCAN1 could be a potential therapeutic target in many inflammatory and autoimmune diseases with dysregulation of TLR signaling.

## Supporting information

S1 Fig *Rcan1-4* transcription is induced by *P*. *aeruginosa* LPS in macrophages. Wild-type (+/+) BMMs were treated with 200 ng/ml *P*. *aeruginosa* LPS for 1 h, 2 h, 4 h or left untreated (NT). Total RNA isolated from these cells was reverse transcribed to cDNA and subjected to real-time quantitative PCR for *Rcan1-1* and *Rcan1-4*. The *Rcan1-1* and *Rcan1-4* gene expression was normalized to housekeeping control gene *HPRT* (n = 3 ± SEM, *p<0.05).
(TIFF)

S2 Fig RCAN1-deficient BMMs display enhanced cytokine gene expression in MyD88-dependent pathway and reduced cytokine gene expression in TRIF-dependent pathway during *P*. *aeruginosa* LPS stimulation. Wild type (+/+) and RCAN1-deficient (-/-) BMMs were treated with 200 ng/ml *P*. *aeruginosa* LPS for 1 h, 2 h, 4 h or left untreated (NT). Total RNA isolated from these cells was reverse transcribed to cDNA and subjected to real-time quantitative PCR for determining *IL-6* (A), *TNF* (B), *MIP2* (C) *IFN-β* (D), *RANTES* (E) and *IP-10* (F) gene expression. The gene expression was normalized to housekeeping control gene *HPRT* (n = 3 ± SEM, *p<0.05, ***p<0.001 ****p<0.0001).
(TIFF)

S3 Fig RCAN1 deficiency upregulates MyD88-mediated cytokine and chemokine production but downregulates TRIF-IRF-ISRE-mediated cytokine and chemokine production in BALF alveolar macrophages during *P*. *aeruginosa* LPS stimulation. Wild-type (+/+) and RCAN1-deficient (-/-) alveolar macrophages were stimulated with 200 ng/ml *P*. *aeruginosa* LPS for 6 h or left untreated (NT). Cell supernatants were collected for the determination of IL-6 (A), TNF (B), MIP2 (C), IFNβ (D), RANTES (E) and IP-10 (F) secretion by ELISA. (n = 3 ± SEM, *p<0.05).
(TIFF)

S4 Fig RCAN1 differentially regulates MAPK kinase activation *in vitro* in response to *P*. *aeruginosa* LPS challenge. Wild-type (+/+) and RCAN1-deficient (-/-) BMMs were challenged with 200 ng/ml *P*. *aeruginosa* LPS for 3 h, 6 h, 12 h and 24 h or left untreated (NT). Cell lysates were subjected to Western blot analysis for phospho- and total ERK, JNK and p38, as well as actin as loading control. Blots are representative of three independent experiments (A). Densitometry analysis of phosphorylated ERK (B), JNK (C) and p38 (D) was normalized to their total protein respectively (n = 3 ± SEM, *p<0.05, **p<0.01).
(TIFF)

S5 Fig RCAN1 deficiency does not significantly impair IRF7 mRNA expression in lung in response to *P*. *aeruginosa* LPS stimulation. Wild-type (+/+) and RCAN1-deficient (-/-) mice were administered intranasally with 1 μg *P*. *aeruginosa* LPS per gram of body weight, or an equivalent volume of saline as a control (NT) for 4 h. The total RNA extracted from lungs was reverse transcribed to cDNA and subjected to real-time quantitative PCR for *IRF3* (A) and *IRF7* (B) gene expression. The gene expression was normalized to housekeeping control gene *HPRT* (n = 3 ± SEM).
(TIFF)

S6 Fig RCAN1-deficient mice display enhanced neutrophil infiltration in lung following *P*. *aeruginosa* LPS stimulation. Wild-type (+/+) and RCAN1-deficient (-/-) mice were stimulated intranasally with 1 μg *P*. *aeruginosa* LPS per gram of body weight, or an equivalent volume of saline as a control (NT) for 4 h or 24 h. Lungs and BALF were collected after 4 h or 24 h. MPO activities were measured in the Lung (A) and BALF (B) lysate (n = 9 ± SEM, ***p<0.001). The upper lobe of the left lung was collected for H&E staining (original magnification X 20 or X 100) (C). Pictures are representative of 6 mice.
(TIFF)

S7 Fig Schematic representation of RCAN1-regulated MyD88- and TRIF-dependent signaling pathways. Binding of *P*. *aeruginosa* LPS to TLR4 activates MyD88- and TRIF-dependent signaling pathways. RCAN1 downregulates MyD88-NF-κB pathway through inhibition of IκBα phosphorylation, and promotes activation of TRIF-ISRE pathway through regulation of IRF7 activation and expression.
(TIFF)
