# Supplementary figures and images for: Regulator of calcineurin 1 differentially regulates TLR-dependent MyD88 and TRIF signaling pathways
Source: PLoS One. 2018 May 25;13(5):e0197491. doi: 10.1371/journal.pone.0197491 (PMC5969770; doi:10.1371/journal.pone.0197491)

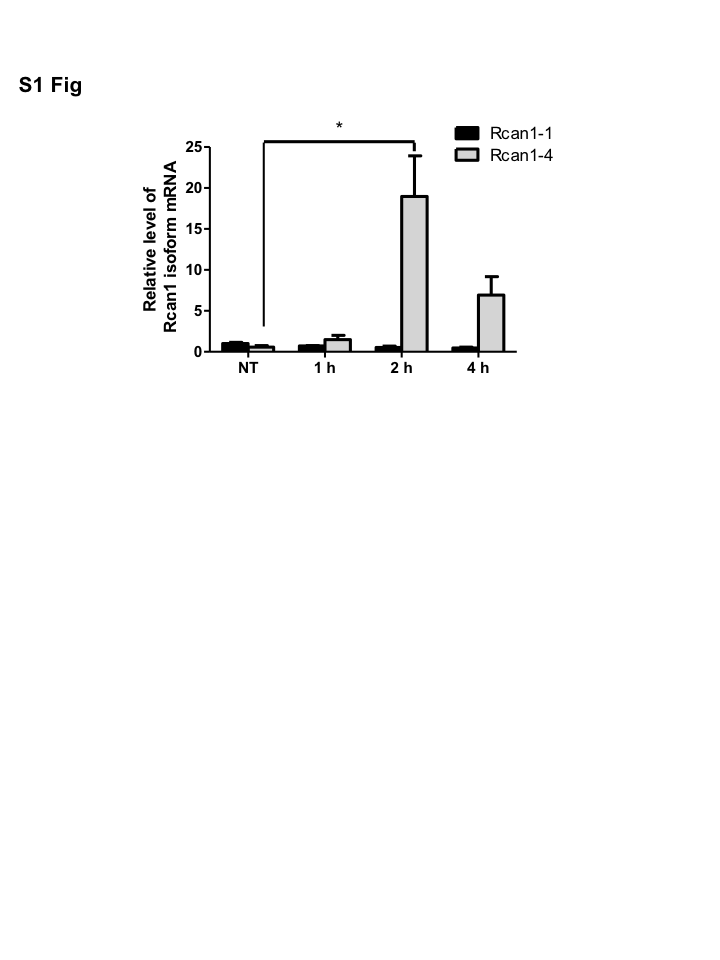

Supplement: S1 Fig — Wild-type (+/+) BMMs were treated with 200 ng/ml P. aeruginosa LPS for 1 h, 2 h, 4 h or left untreated (NT). Total RNA isolated from these cells was reverse transcribed to cDNA and subjected to real-time quantitative PCR for Rcan1-1 and Rcan1-4. The Rcan1-1 and Rcan1-4 gene expression was normalized to housekeeping control gene HPRT (n = 3 ± SEM, *p<0.05). (TIFF) [file pone.0197491.s001.tiff]

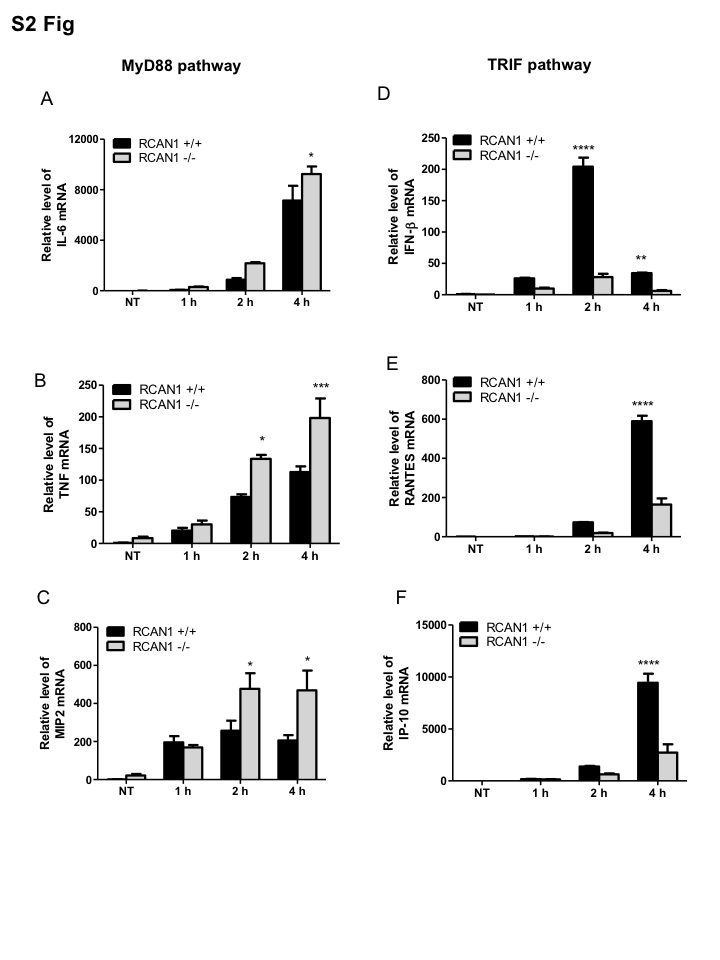

Supplement: S2 Fig — Wild type (+/+) and RCAN1-deficient (-/-) BMMs were treated with 200 ng/ml P. aeruginosa LPS for 1 h, 2 h, 4 h or left untreated (NT). Total RNA isolated from these cells was reverse transcribed to cDNA and subjected to real-time quantitative PCR for determining IL-6 (A), TNF (B), MIP2 (C) IFN-β (D), RANTES (E) and IP-10 (F) gene expression. The gene expression was normalized to housekeeping control gene HPRT (n = 3 ± SEM, *p<0.05, ***p<0.001 ****p<0.0001). (TIFF) [file pone.0197491.s002.tiff]

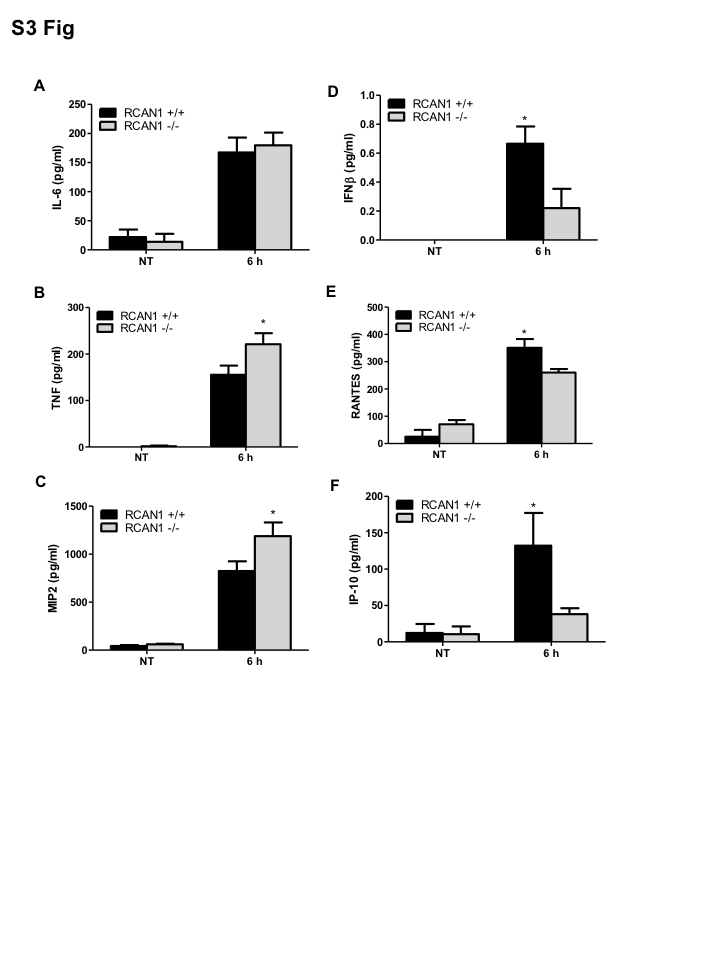

Supplement: S3 Fig — Wild-type (+/+) and RCAN1-deficient (-/-) alveolar macrophages were stimulated with 200 ng/ml P. aeruginosa LPS for 6 h or left untreated (NT). Cell supernatants were collected for the determination of IL-6 (A), TNF (B), MIP2 (C), IFNβ (D), RANTES (E) and IP-10 (F) secretion by ELISA. (n = 3 ± SEM, *p<0.05). (TIFF) [file pone.0197491.s003.tiff]

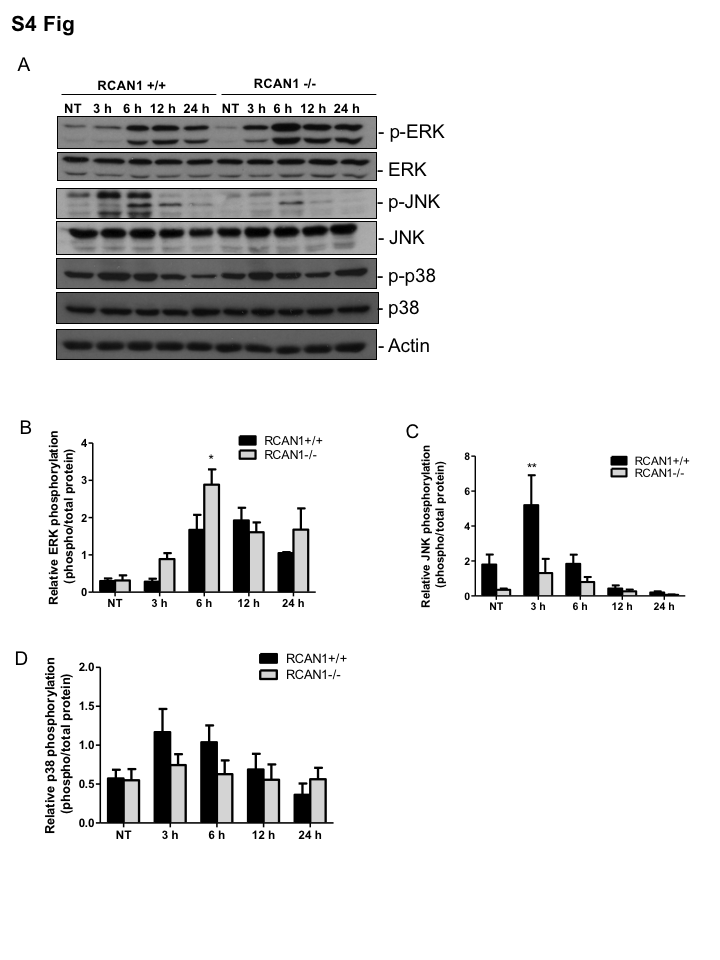

Supplement: S4 Fig — Wild-type (+/+) and RCAN1-deficient (-/-) BMMs were challenged with 200 ng/ml P. aeruginosa LPS for 3 h, 6 h, 12 h and 24 h or left untreated (NT). Cell lysates were subjected to Western blot analysis for phospho- and total ERK, JNK and p38, as well as actin as loading control. Blots are representative of three independent experiments (A). Densitometry analysis of phosphorylated ERK (B), JNK (C) and p38 (D) was normalized to their total protein respectively (n = 3 ± SEM, *p<0.05, **p<0.01). (TIFF) [file pone.0197491.s004.tiff]

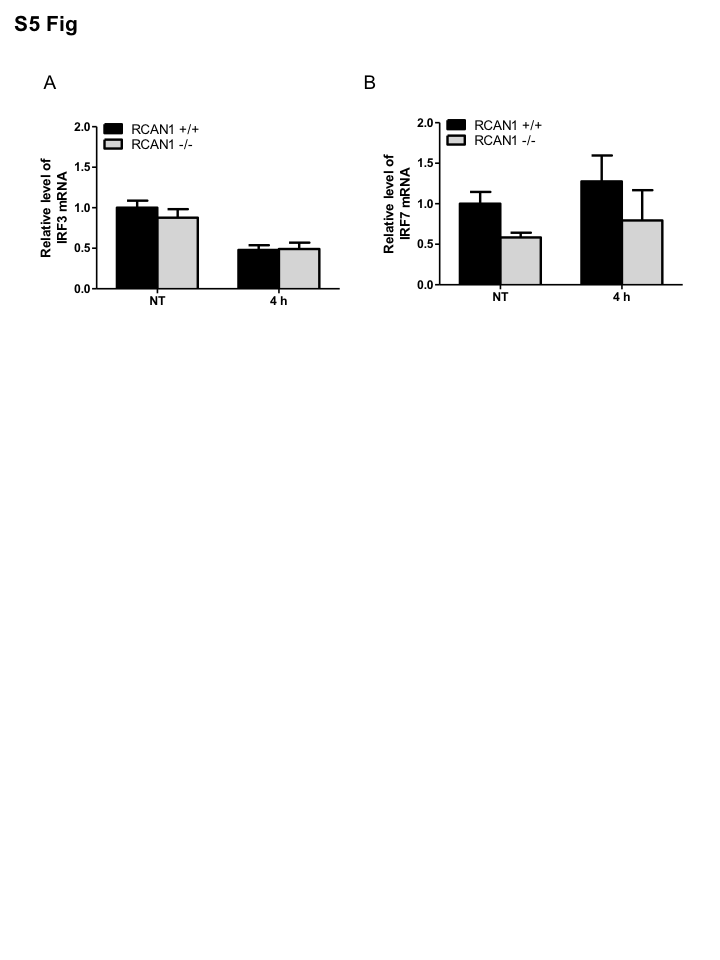

Supplement: S5 Fig — Wild-type (+/+) and RCAN1-deficient (-/-) mice were administered intranasally with 1 μg P. aeruginosa LPS per gram of body weight, or an equivalent volume of saline as a control (NT) for 4 h. The total RNA extracted from lungs was reverse transcribed to cDNA and subjected to real-time quantitative PCR for IRF3 (A) and IRF7 (B) gene expression. The gene expression was normalized to housekeeping control gene HPRT (n = 3 ± SEM). (TIFF) [file pone.0197491.s005.tiff]

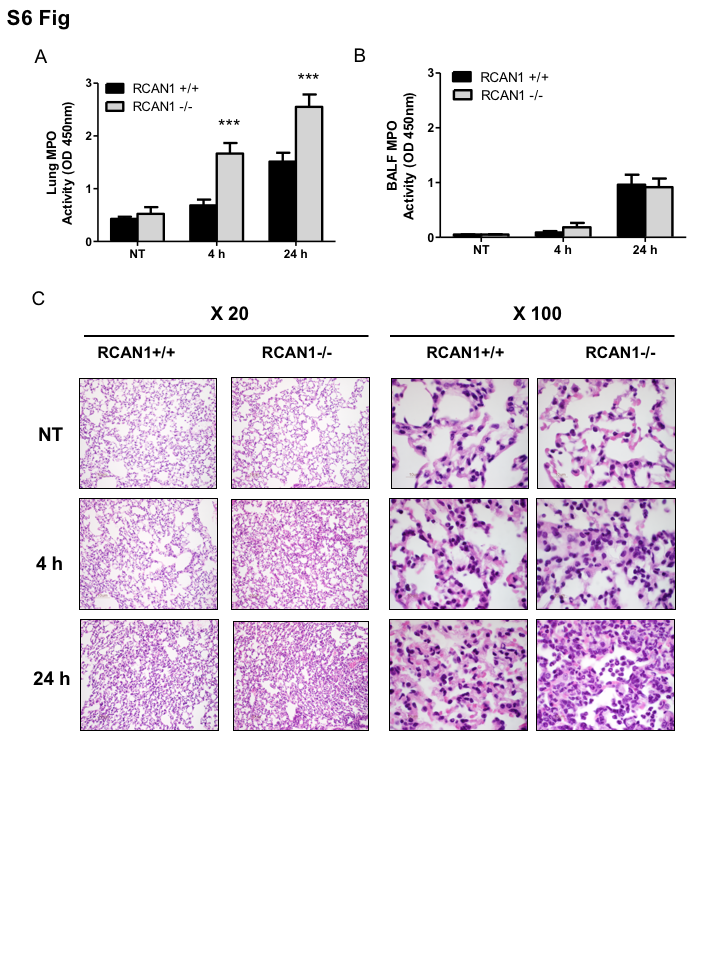

Supplement: S6 Fig — Wild-type (+/+) and RCAN1-deficient (-/-) mice were stimulated intranasally with 1 μg P. aeruginosa LPS per gram of body weight, or an equivalent volume of saline as a control (NT) for 4 h or 24 h. Lungs and BALF were collected after 4 h or 24 h. MPO activities were measured in the Lung (A) and BALF (B) lysate (n = 9 ± SEM, ***p<0.001). The upper lobe of the left lung was collected for H&E staining (original magnification X 20 or X 100) (C). Pictures are representative of 6 mice. (TIFF) [file pone.0197491.s006.tiff]

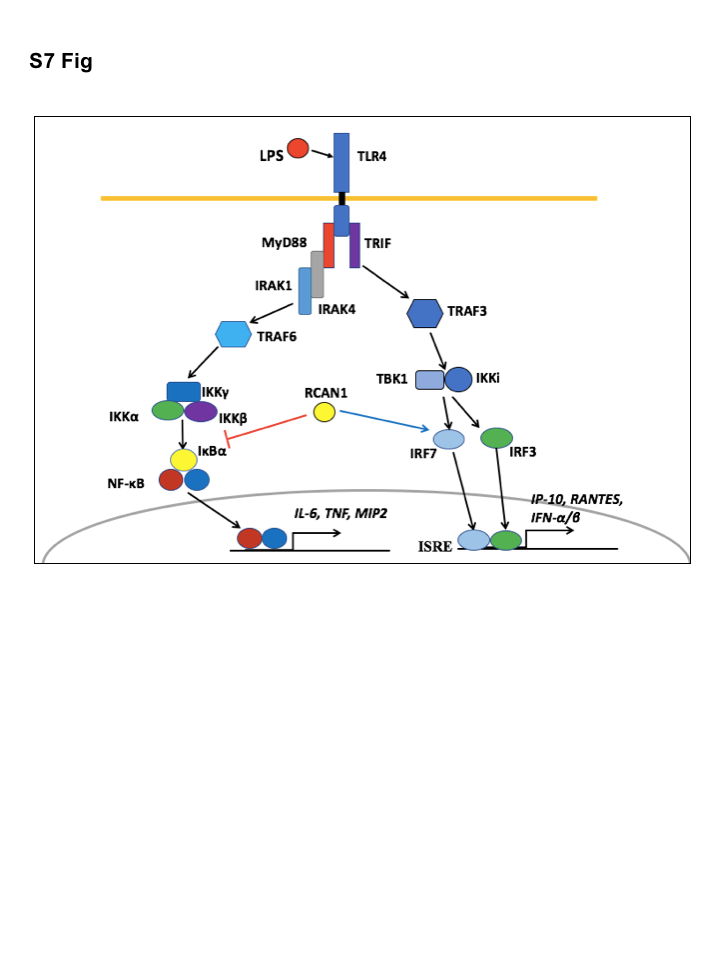

Supplement: S7 Fig — Binding of P. aeruginosa LPS to TLR4 activates MyD88- and TRIF-dependent signaling pathways. RCAN1 downregulates MyD88-NF-κB pathway through inhibition of IκBα phosphorylation, and promotes activation of TRIF-ISRE pathway through regulation of IRF7 activation and expression. (TIFF) [file pone.0197491.s007.tiff]
